# Cultural orientation, power, belief in conspiracy theories, and intentions to reduce the spread of COVID‐19

**DOI:** 10.1111/bjso.12397

**Published:** 2020-06-27

**Authors:** Mikey Biddlestone, Ricky Green, Karen M. Douglas

**Affiliations:** ^1^ University of Kent UK

**Keywords:** Individualism, collectivism, COVID‐19, conspiracy theories, powerlessness

## Abstract

The current study investigated cultural and psychological factors associated with intentions to reduce the spread of COVID‐19. Participants (*n* = 704) completed measures of individualism–collectivism, belief in conspiracy theories about COVID‐19, feelings of powerlessness, and intentions to engage in behaviours that reduce the spread of COVID‐19. Results revealed that vertical individualism negatively predicted intentions to engage in social distancing, directly and indirectly through both belief in COVID‐19 conspiracy theories and feelings of powerlessness. Vertical collectivism positively predicted social distancing intentions directly. Horizontal collectivism positively predicted social distancing intentions indirectly through feelings of powerlessness. Finally, horizontal collectivism positively predicted hygiene‐related intentions both directly and indirectly through lower feelings of powerlessness. These findings suggest that promoting collectivism may be a way to increase engagement with efforts to reduce the spread of COVID‐19. They also highlight the importance of examining the interplay between culture and both personal feelings (powerlessness) and information consumption (conspiracy theories) during times of crisis.

## Background

In March 2020, the global COVID‐19 crisis was officially labelled a pandemic (WHO, 2020a). Governments around the world have since taken unprecedented steps to mitigate the spread of the virus, such as encouraging social distancing and improving hygiene practices. Making these measures work is a significant challenge for governments (Tominey, 2020). It is therefore important to understand what factors influence people’s intentions to comply. Researchers have emphasized the importance of considering social psychological factors in attempts to limit the spread of COVID‐19 (Van Bavel et al., 2020). Drawing on social psychological expertise, advisory groups have specifically highlighted the role that cultural factors could play in people’s intentions to engage with government instructions (SAGE, 2020). In the current research, we examine the link between cultural orientation and people’s intentions to engage in behaviours to reduce the spread of COVID‐19. We further examine two potential mediators of this relationship, namely conspiracy theories surrounding COVID‐19 (Sweney & Waterson, 2020) and feelings of powerlessness (Xiang et al., 2019; Jolley & Douglas, 2014a, 2014b).

### Culture and intentions to reduce the spread of COVID‐19

Cultural psychology has paid particular attention to the dimensions of individualism, which focuses on prioritizing individual needs above the group’s, and collectivism, which focuses on prioritizing group needs above the individual’s (see Hofstede *et al*., 2010). Research has consistently shown that collectivist (versus individualist) cultures demonstrate greater compliance and adherence to social norms (e.g., Kim *et al*., 1994). In collectivist societies, it is ideal to meet social duties and obligations to maintain group harmony (Miller, Bersoff & Harwood, 1990), whereas individualist societies tend to promote personal freedom over harmony (Markus & Kitayama, 1991). These cultural orientations can also be measured at the level of the individual – that is, within a society some people have a more individualist (versus collectivist) orientation than others (Triandis & Singelis, 1998). This is further broken down into vertical individualism (viewing the self as an autonomous individual who accepts inequality), horizontal individualism (viewing the self as an autonomous individual who prefers equality), vertical collectivism (viewing the self as part of a collective whilst accepting inequality), and horizontal collectivism (viewing the self as part of a collective that emphasizes equality; Singelis *et al*., 1995).

Adherence to social norms is an important response to collective crises (see Murray *et al*., 2011; see also Atran, 2012, for different perspectives). Following the COVID‐19 pandemic, behavioural science advisors in the United Kingdom have emphasized that promoting a sense of collectivism in individuals could reduce instances of public disorder, increase self‐policing, and promote social norms about what constitutes appropriate pandemic behaviour (SAGE, 2020). A sense of collectivism may improve people’s attitudes towards actions that involve personal sacrifices. Supporting this idea, at the cultural level Gelfand et al. (2020) found that tighter cultures (considered more collectivist; e.g., Hong Kong, Taiwan, and South Korea) have been able to contain the spread of the virus more efficiently and effectively than looser cultures (considered more individualist; e.g., the United States, Spain, and Italy).

Further indirect evidence points to the potential importance of individualism–collectivism in predicting intentions to reduce the spread of COVID‐19. Specifically, research examining climate change perceptions has demonstrated that individualists are less likely than collectivists to engage in climate‐friendly actions that involve personal sacrifice (Xiang *et al*., 2019). Considering these findings, we expect that individualism will have negative, and collectivism will have positive, implications for adherence to behaviours aimed at reducing the spread of COVID‐19.

We examined two potential mediators of this predicted relationship. Specifically, we investigated the extent to which belief in conspiracy theories and feelings of powerlessness mediate the relationship between cultural orientation and intentions to engage in behaviours that reduce the spread of COVID‐19.

### Conspiracy theories

Conspiracy theories have been a prominent feature of the pandemic, from the notion that China manufactured the disease as a bioweapon to use against the west, to the idea that 5G technologies spread it (van Bavel *et al*., 2020). Although no research to date directly links belief in conspiracy theories to individualism or collectivism, there is much indirect evidence suggesting that they should be related. For example, the need for uniqueness, which is argued to be a central feature of individualism (e.g., Cai *et al*., 2018; Wang, Fan, & Ouyang, 2017), consistently predicts conspiracy beliefs (Hart & Graether, 2018; Lantian *et al*., 2017). Furthermore, individualists (versus collectivists) score higher on the Dark Triad (narcissism, Machiavellianism, and psychopathy; Wilson & Hartley, 2015), and these traits also consistently predict conspiracy beliefs (Cichocka *et al.*, 2015; Douglas & Sutton, 2011; March & Springer, 2019). Therefore, it is reasonable to expect that individualism will be positively, and collectivism negatively, associated with belief in conspiracy theories.

In turn, belief in conspiracy theories has important social and health‐related consequences (Douglas *et al*., 2019) including reduced engagement with mainstream politics, climate change initiatives, and vaccination programmes (Jolley & Douglas, 2014a, 2014b) and has been identified as a likely obstacle to constructive public responses to the pandemic (Van Bavel *et al*., 2020). Drawing this research together, we therefore predict that individualists (versus collectivists) will show decreased compliance with COVID‐19 mitigating activities as a consequence of their belief in COVID‐19 conspiracy theories.

### Powerlessness

Cultural orientation is also associated with feelings of powerlessness – the sense of being unable to make a meaningful impact on important issues (Xiang *et al*., 2019). For example, Xiang et al. found that individualism was positively – and collectivism was negatively – related to feelings of powerlessness concerning climate change. This suggests that collectivists may garner feelings of power to overcome issues that require collective effort, whereas individualists may be less able to draw on such power. That is, collectivists may feel that they can rely on others to take action on global issues, making their own personal contribution in such cases more meaningful. Individualists, on the other hand, are less likely to feel empowered by collective support, ultimately making their own personal contribution feel less meaningful. Therefore, it is possible that feelings of powerlessness may help explain individualists’ and collectivists’ different responses to COVID‐19.

Research has also demonstrated that feelings of powerlessness predict health‐related behaviours. For example, Jolley and Douglas (2014b) found that feelings of powerlessness mediated the relationship between conspiracy beliefs and lower vaccination intentions, a relationship they also demonstrated for climate‐friendly behaviours (Jolley & Douglas, 2014a). Feelings of powerlessness are therefore also likely to negatively influence people’s responses to the pandemic. Drawing this research together, we therefore explore the possibility that individualists (versus collectivists) will show decreased compliance with COVID‐19 mitigating activities as a consequence of heightened feelings of powerlessness. Following previous research, we also predict that feelings of powerlessness will mediate the relationship between conspiracy belief and COVID‐19 intentions.

### The present study

We tested the relationships between individualism–collectivism and behavioural intentions during the COVID‐19 pandemic. These intentions were specifically related to social distancing and hygiene measures. We also examined the potential mediating roles of conspiracy beliefs and powerlessness. We hypothesized that both vertical and horizontal individualism would predict lower, and that both vertical and horizontal collectivism would predict higher, intentions to engage in behaviours that reduce the spread of COVID‐19. Furthermore, we hypothesized that the relationships between individualism–collectivism and engagement with behaviours that reduce the spread of COVID‐19 would be mediated by both belief in COVID‐19 conspiracy theories and feelings of powerlessness. Finally, we expected to conceptually replicate previous findings by showing that feelings of powerlessness mediate the relationship between COVID‐19 conspiracy beliefs and COVID‐19 intentions.^1^We included additional variables and hypotheses but omit them here due to space restrictions. The key pre‐registered hypothesis (i.e., that vertical and horizontal individualism would predict lower, and that vertical and horizontal collectivism would predict higher, COVID‐19 intentions) is presented here. Two of the pre‐registered mediation hypotheses (i.e., that relationships between individualism–collectivism and COVID‐19 intentions would be mediated by both conspiracy beliefs and feelings of powerlessness, and that feelings of powerlessness would mediate the relationship between conspiracy beliefs and intentions) are also presented here. Pre‐registration documentation is here: https://osf.io/sqtpz/?view_only=cb1a947ac12a41ec8e35c878df76c1a4, and other pre‐registered analyses are presented in the Supplementary Materials.


## Method

### Participants and design

Seven hundred and twenty‐four participants^2^According to Fritz and MacKinnon (2007), to detect a small mediation effect with a power of .8 using bias‐corrected bootstrapping, a minimum sample size of 462 is required. were recruited for this correlational study (from 4 to 13 April 2020) via posts on social media (*n* = 413) and Reddit forums (*n* = 311).^3^Controlling for method of data collection did not affect the pattern of results (see Supplementary Materials). We excluded participants who failed at least one of two attention checks (*n* = 20). The remaining participants (*n* = 704; 376 women, 306 men, 10 non‐binary, 10 rather not say, 2 transgender, *M*_age_ = 37.26 years, *SD*_age_ = 12.51)^4^Age is based on *n* = 701. were included in the final analyses. Of this sample, 34.2% were British, 30.4% were American, and the remaining 35.4% were made up of 64 different nationalities.^5^Nationality is based on *n* = 694.


### Materials and procedure

The questionnaire was designed and administered using Qualtrics. Participants read an information page and gave their consent to take part. They were then asked to complete a series of measures in random order, except for the demographic measures which always appeared in the same order at the end of the questionnaire.

To measure intentions to reduce the spread of COVID‐19, participants reported the likelihood that they would engage in 12 behaviours. Eight were related to social distancing (e.g., ‘Remain at least 2 metres (6 feet) apart from other people’; ‘Isolate yourself for at least 1 week if you show even mild cold or flu symptoms’; 1 = *definitely not* to 5 = *definitely yes*, α = .73) and four were related to hygiene measures (e.g., ‘Wash your hands after every outing’; ‘Wash your hands before eating’; α = .55). Individualism–collectivism was measured using Triandis and Gelfand’s (1998) scale. Participants rated the extent to which 16 items described them. Four items measured vertical individualism (e.g., ‘It is important that I do my job better than others’ and ‘Winning is everything’; 1 = *definitely no* to 5 = *definitely yes,* α = .65), four measured horizontal individualism (e.g., ‘I’d rather depend on myself than others’ and ‘My personal identity, independent of others, is very important to me’; α = .64), four measured vertical collectivism (e.g., ‘It is important to me that I respect the decisions made by my groups’ and ‘Parents and children must stay together as much as possible’; α = .64), and four measured horizontal collectivism (e.g., ‘I feel good when I cooperate with others’ and ‘To me, pleasure is spending time with others’; α = .69).

We designed 10 statements measuring belief in COVID‐19 conspiracy theories (e.g., ‘Coronavirus was purposefully created in, and released from, a biochemistry lab in Wuhan, China’ and ‘The implementation of 5G technology is a means of deliberately spreading Coronavirus’; 1 = *strongly disagree* to 7 = *strongly agree*, α = .90). A three‐item scale of powerlessness concerning the spread of COVID‐19 was adapted from Jolley and Douglas (2014a, 2014b; e.g., ‘I feel that the Coronavirus is too big for my actions to have an impact’ and ‘I feel that my actions will not affect the outcome of Coronavirus’; 1 = *strongly disagree* and 6 = *strongly agree*, α = .88).

Lastly, participants provided demographic details, which we included as control variables. In addition to age and gender, they rated their education level (1 = *high (senior) school/lower*, 2 = *bachelor’s,* 3 = *master’s*, 4 = *post‐graduate/higher*), religiosity (1 = *not religious at all*, 7 = *very religious*), and political orientation (1 = *extremely left‐wing,* 5 = *extremely right‐wing*). We also controlled for whether participants had any underlying health conditions that may impact the severity of symptoms if they contracted COVID‐19 (*yes* or *no*). Finally, participants were debriefed and thanked.

## Results

Means, standard deviations, and zero‐order correlations are in Table 1. To test our hypotheses, we used the *Lavaan* package in *R* to perform confirmatory factor analysis (CFA) and structural equation modelling (SEM). Inspection of normality statistics showed that the data for a number of items in the intentions and conspiracy belief scales were skewed. Therefore, bootstrapping was performed on all subsequent analyses.

**Table 1:** Means, standard deviations, and zero‐order correlations

Variable	*M*	*SD*	1	2	3	4	5	6	7	8
1. Hygiene intentions	3.85	0.63	–	.35***	–.10**	.02	.08*	.15***	–.02	–.24***
2. Social distancing intentions	4.31	0.65		–	–.14***	–.04	.08*	.18***	–.16***	–.26***
3. Vertical individualism	4.24	1.52			–	.23***	.17***	–.16***	.14***	.23***
4. Horizontal individualism	6.61	1.33				–	–.04	–.14***	.08*	.12**
5. Vertical collectivism	6.15	1.49					–	.32***	.09*	–.04
6. Horizontal collectivism	6.75	1.31						–	–.03	–.26***
7. COVID‐19 conspiracies	1.50	0.57							–	.18***
8. Powerlessness	2.46	1.20								–

Note

*p < .05; **p < .01; ***p < .001.

**Figure 1:**
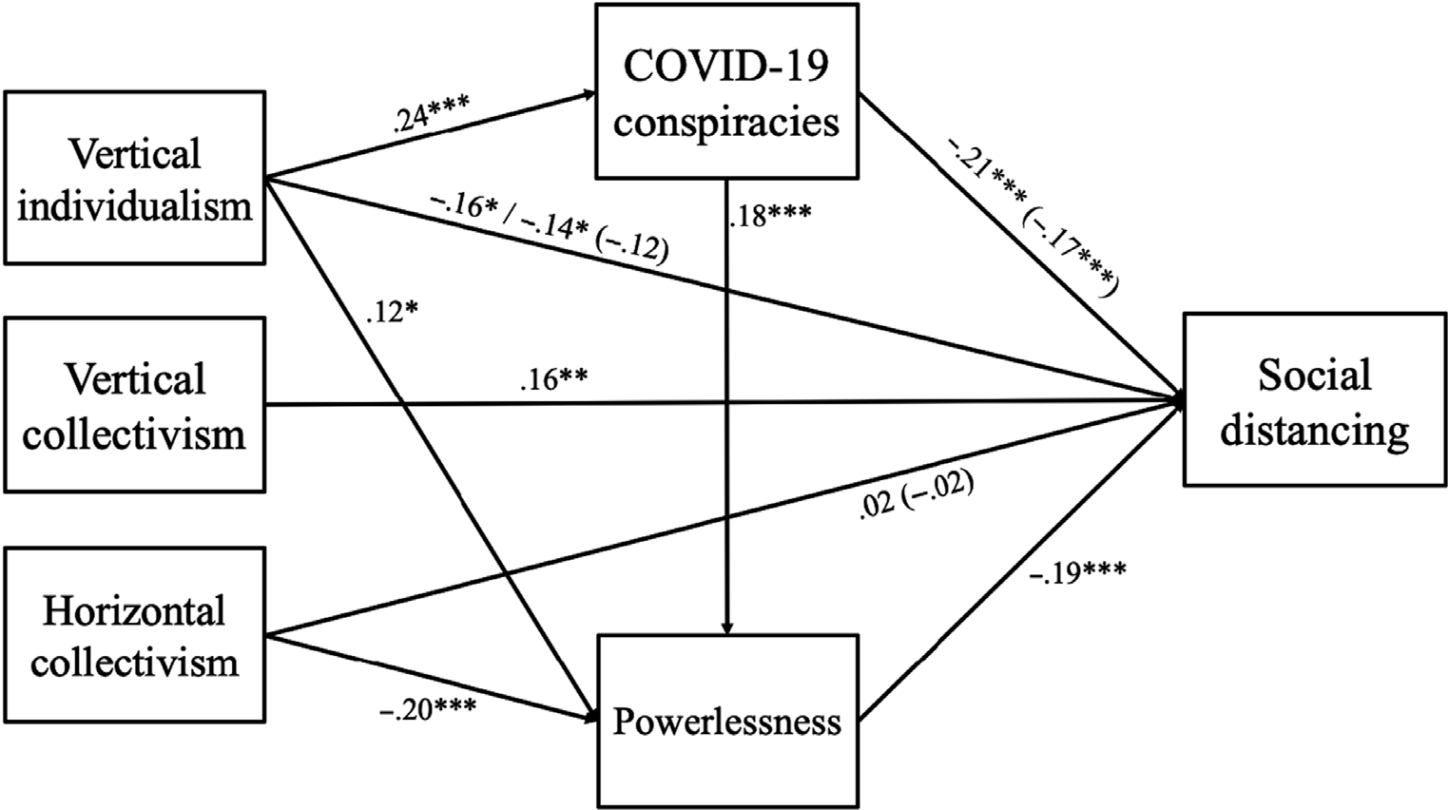
Predictors of social distancing intentions in the final SEM model (controlling for demographics). Non‐significant paths, demographic paths, and paths predicting hygiene intentions have been removed for ease of viewing. Direct effects are reported in parentheses, and total effects are reported without parentheses. The total effect of vertical individualism on social distancing is firstly reported through conspiracy beliefs, and secondly through powerlessness. All values are standardised beta coefficients.

After creating CFA models for each of our independent variables, potential mediators, and dependent variables, we performed SEM controlling for demographic variables^6^So that we could include gender as a dichotomous variable in the analysis, we excluded participants who did not report being male or female (*n* = 22; remaining *n* = 682). and using the factors from the CFA, achieving reasonable overall global and local fit, χ^2^(904) = 2015.05, *p *< .001; *CFI* = .89; *TLI* = .88; *RMSEA* = .04, *p* = 1; *SRMR* = .06 (see Figure 1).^7^Due to skewness and kurtosis, one item from the social distancing measure was removed (‘Isolate yourself for at least 1 week if you know you have been in contact with someone with Coronavirus’) and one hygiene item was also removed (‘Wash your hands after using the toilet’). This was not problematic conceptually since similar items were already included in the analyses. The final sub‐scales provided the best model fit, and reliability was not compromised by removing these items (social distancing = .73, hygiene = .52). Although this final model did not quite achieve good global fit, we stopped adding paths recommended by the modification indices when adding said paths started worsening the model fit.

**Figure 2:**
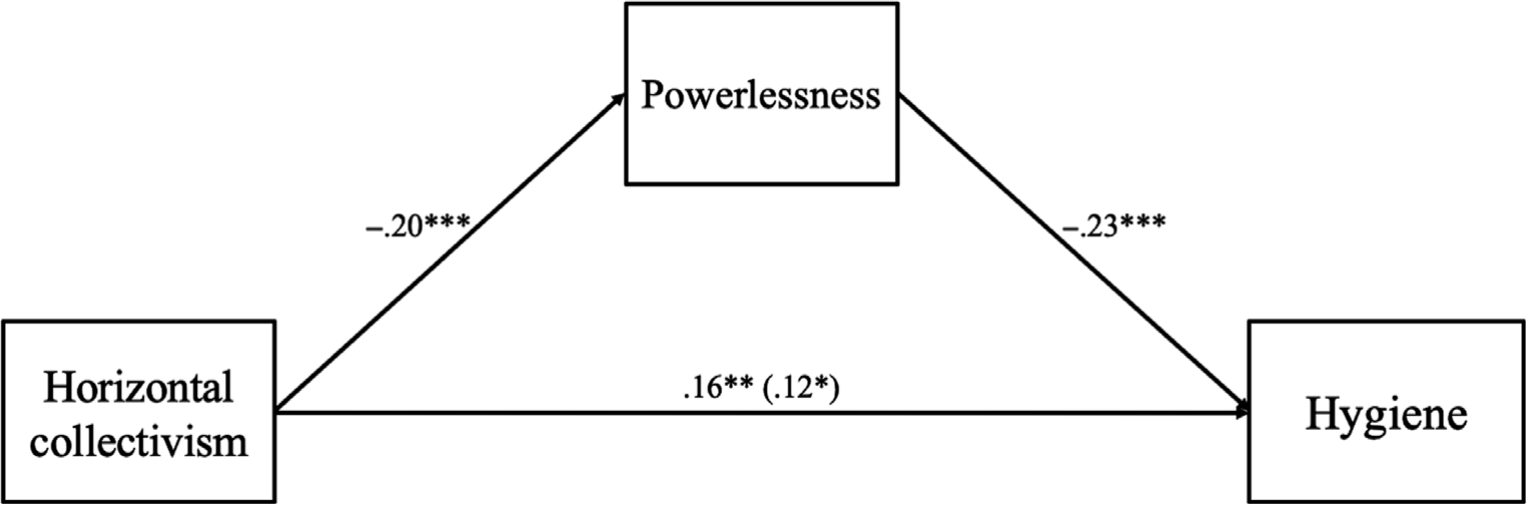
Predictors of hygiene intentions in the final SEM model (controlling for demographics). Non‐significant paths, demographic paths, and paths predicting social distancing intentions have been removed for ease of viewing. Direct effects are reported in parentheses, and total effects are reported without parentheses. All values are standardised beta coefficients.

In the model, social distancing intentions were positively predicted by horizontal collectivism, but only indirectly through lower feelings of powerlessness, standardized indirect effect = .04 [0.02, 0.06] (see Figure 1). Vertical individualism, however, negatively predicted social distancing intentions, directly and indirectly through belief in COVID‐19 conspiracy theories, standardized indirect effect = −.04 [−0.06, −0.02], and feelings of powerlessness, standardized indirect effect = −0.02 [−0.04, −0.01]. Vertical collectivism positively predicted social distancing on a direct path alone. Hygiene intentions were only positively predicted by horizontal collectivism, both directly and indirectly through lower feelings of powerlessness, standardized indirect effect = .05 [0.02, 0.07] (see Figure 2).^8^Results remained largely the same when controlling for nationality (see Supplementary Materials). Finally, as predicted, belief in COVID‐19 conspiracy theories negatively predicted social distancing intentions both directly and indirectly through feelings of powerlessness, standardized indirect effect = −.04 [−0.05, −0.01].^9^Model fit was less satisfactory without the link between COVID‐19 conspiracy belief and feelings of powerlessness. See Supplementary Materials for the alternative model.


## Discussion

Individualists and collectivists showed different intentions to engage in activities that reduce the spread of COVID‐19. Specifically, vertical individualism directly predicted lower intentions to engage in social distancing behaviours once the mediating effects were taken into account. Levels of collectivism (both horizontal and vertical), on the other hand, predicted higher social distancing intentions. Furthermore, horizontal collectivism positively predicted hygiene‐related intentions, but individualism was not associated with these behaviours. Interestingly, support for our hypotheses was stronger for the social distancing than hygiene intentions. However, supporting Gelfand et al.'s (2020) analysis, the current research broadly suggests that a ‘tighter’ cultural orientation is associated with positive responses to the pandemic and that government efforts to improve compliance could therefore consider promoting a collectivist orientation amongst the general public.

The current research also adds to existing findings by demonstrating that individualist–collectivist differences in response to COVID‐19 were further influenced by feelings of personal power. In addition, vertical individualists (but not collectivists) appear to be affected by the conspiracy theories that are circulating about COVID‐19. The current ‘infodemic’ surrounding COVID‐19 (WHO, 2020b) may also therefore be more likely to affect individualist versus collectivist cultures. Interventions that focus on empowerment and those that address conspiracy theories and other forms of misinformation are therefore likely to improve people’s engagement with behaviours that reduce the spread of COVID‐19.

Our results also support previous findings that collectivists, versus individualists, are more likely to display adaptive responses during times of crisis more generally (e.g., Murray *et al*., 2011; Schaller & Duncan, 2007). The positive link between levels of individualism, powerlessness, and COVID‐19 inaction also corroborates Xiang *et al*.’s (2019) findings in the context of climate change. Moreover, the current findings extend previous literature demonstrating a link between conspiracy belief, feelings of powerlessness, and climate change inaction (see Jolley & Douglas, 2014a, 2014b), by demonstrating that this link may be particularly important for individualists in the case of COVID‐19. Whilst the correlational nature of this study restricts us from making conclusions about cause and effect, previous research suggests that our final mediation models are similar to experimental results in other contexts (see Jolley & Douglas, 2014a, 2014b; Xiang *et al*., 2019). The current findings are therefore likely to be useful beyond the COVID‐19 crisis.

Some other limitations of the current study need to be noted. Despite the large sample size and inclusion of 65 nationalities, almost 65% of the participants were either American or British. The study is therefore not internationally representative and does not allow us to test for cross‐cultural or cross‐national differences. Future research should access collectivist communities to increase the generalizability of these findings. It should also include a general measure of conspiracy belief, such as the Generic Conspiracist Beliefs Scale (GCBS; Brotherton, French, & Pickering, 2013), in order to examine more general links between individualism–collectivism, conspiracy beliefs, powerlessness, and intentions. We also note that effect sizes in the current study were small, suggesting that many other factors influence the COVID‐19 response. Furthermore, the literature requires experimental investigations of the indirect paths observed in our results (Van den Bos et al., 2014). This would allow us to more stringently evaluate behavioural scientists’ recommendation to promote collectivism (SAGE, 2020) as part of the COVID‐19 response.

Overall, the current research provides further insight into the potential role of individualism–collectivism in times of crisis. Clearly communicating that ‘we are all in this together’ could be a fruitful endeavour in encouraging people to comply in this particular case and also during future crises. National leaders would also do well to follow this advice themselves and set an example for the general public. The current research also emphasizes the importance of examining the interplay between cultural factors and personal feelings (powerlessness) and information consumption (conspiracy theories) in this time of crisis. Finally, it raises the interesting possibility that during crises, collectivism encourages a powerful response, but individualism removes a sense of power and replaces it with potentially harmful conspiracy beliefs.

## Author contributions

Mikey Biddlestone, MSc (Conceptualization; Data curation; Formal analysis; Investigation; Methodology; Project administration; Resources; Software; Validation; Visualization; Writing – original draft; Writing – review & editing) Ricky Green (Conceptualization; Data curation; Formal analysis; Investigation; Methodology; Project administration; Software; Validation; Visualization; Writing – original draft; Writing – review & editing) Karen Douglas (Conceptualization; Data curation; Formal analysis; Methodology; Project administration; Supervision; Validation; Writing – review & editing)

## Conflicts of interest

All authors declare no conflict of interest.

## Supporting information


**Appendix S1.** Supplementary materials.

## Data Availability

All data are available via the Open Science Framework: https://osf.io/sqtpz/?view_only=cb1a947ac12a41ec8e35c878df76c1a4. We originally included other variables and hypotheses but do not present them in this paper due to space restrictions. Please see Appendix S1 for these results.
