# Supplementary material for: Cultural orientation, power, belief in conspiracy theories, and intentions to reduce the spread of COVID‐19
Source: Br J Soc Psychol. 2020 Jun 27;59(3):663–73. doi: 10.1111/bjso.12397 (PMC7361833; doi:10.1111/bjso.12397)
Supplement: Supplementary file 1 — Appendix S1. Supplementary materials. [file BJSO-59-663-s001.docx]

**Cultural orientation, powerlessness, belief in conspiracy theories, and intentions to reduce the spread of COVID-19**

**Supplementary Materials**

**Confirmatory Factor Analysis**

Factor loadings and covariance paths from the final model (controlling for demographics) are depicted in the figures below.

(Int = intentions, CT = belief in conspiracy theories, V = vertical, H = horizontal).

***Social distancing***

**
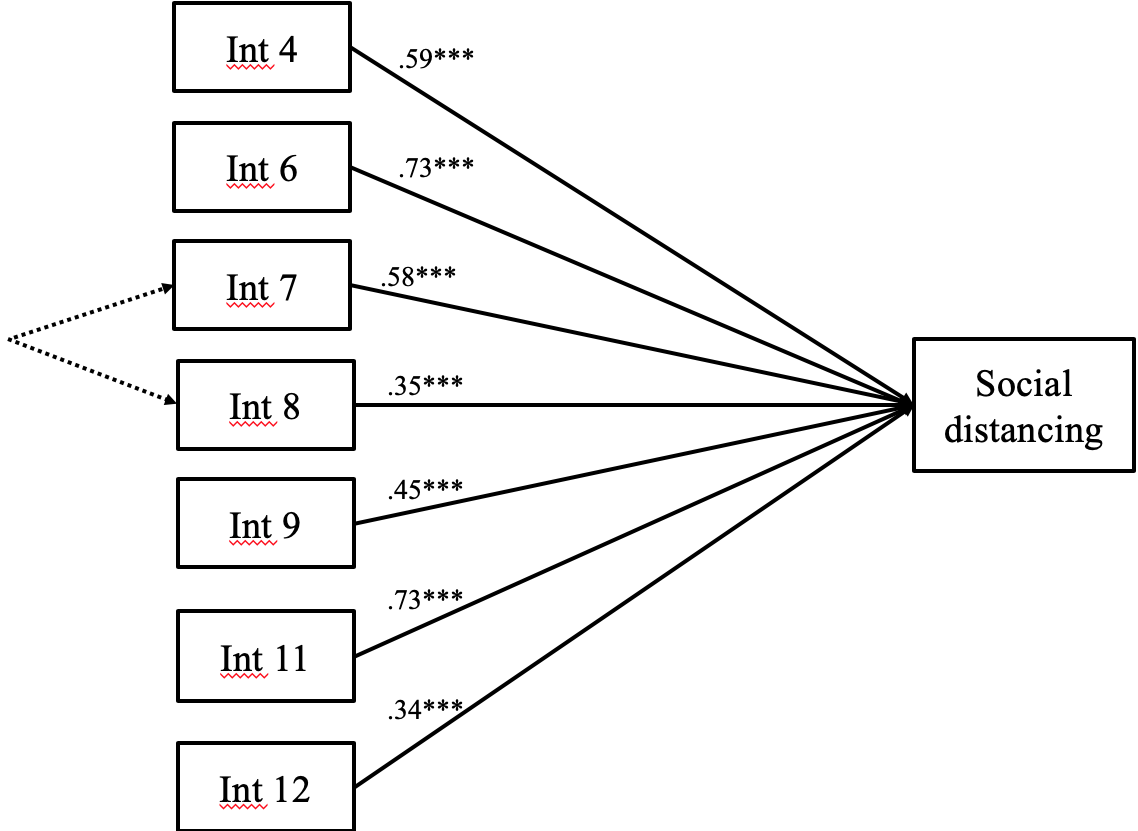
**

There was also covariance between items 5 and 8. This is not shown here.

***Hygiene intentions***


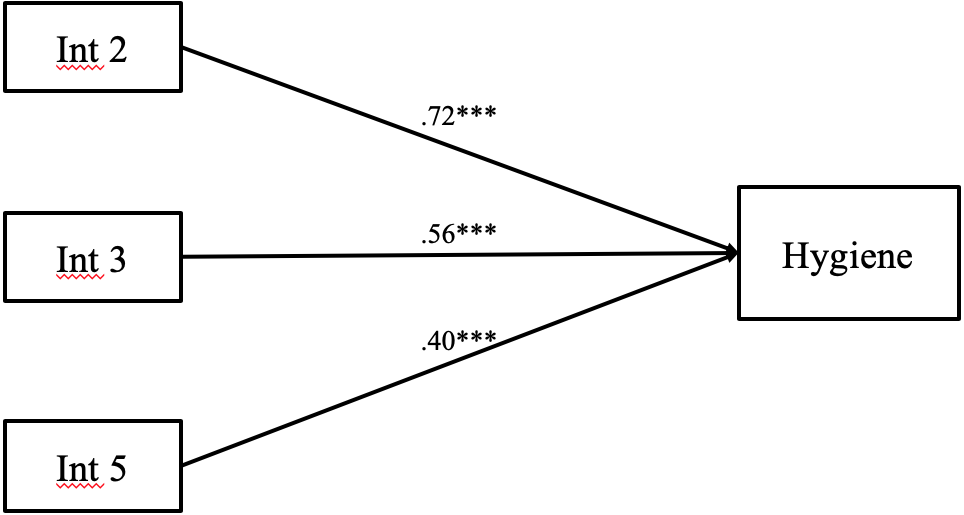


Global fit for intentions CFA, χ^2^(32) = 115.65, *p* < .001; *CFI* = .94; *TLI* = .92; *RMSEA* = .06; *SRMR* = .05.

***Belief in COVID-19 conspiracy theories***


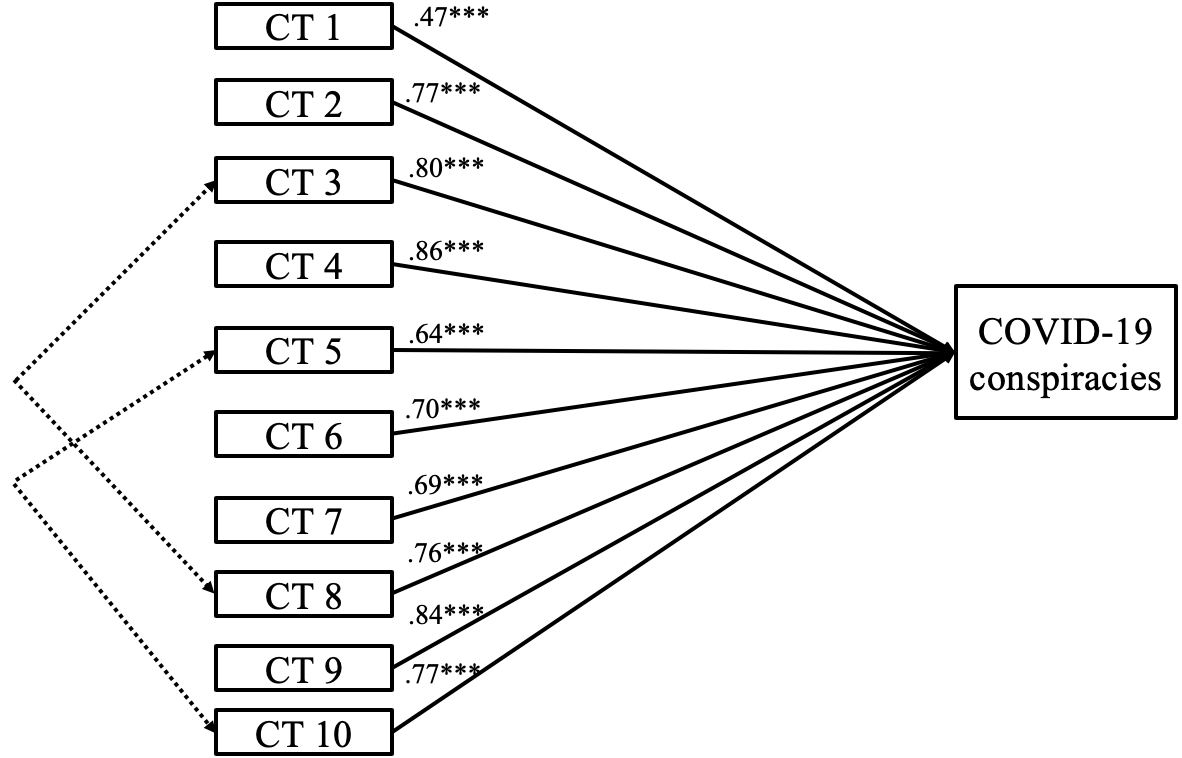


Global fit for belief in conspiracy theories CFA, χ^2^(33) = 178.70, *p* < .001; *CFI* = .97; *TLI* = .95; *RMSEA* = .08; *SRMR* = .04.

***Individualism***

**
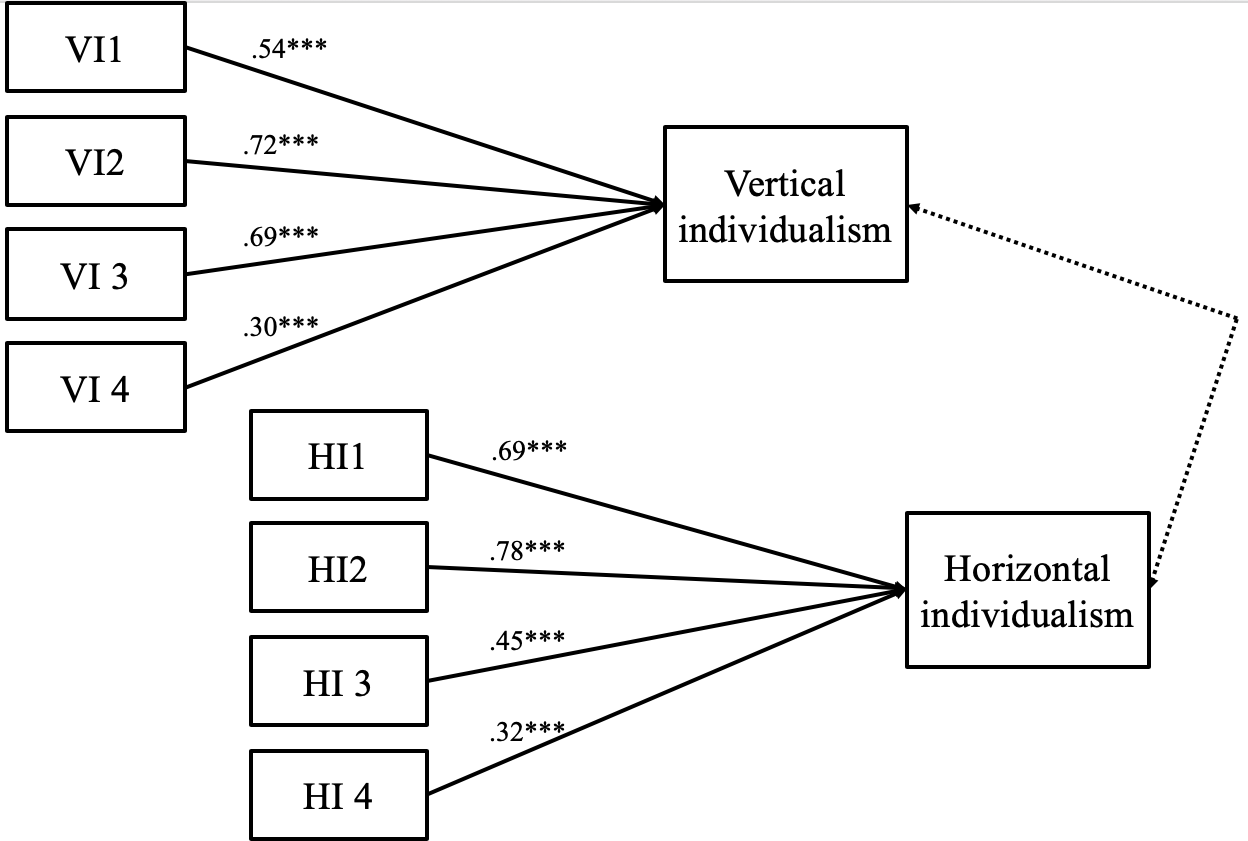
**

Global fit for individualism CFA, χ^2^(19) = 65.14, *p* < .001; *CFI* = .95; *TLI* = .92; *RMSEA* = .06; *SRMR* = .04.

***Collectivism***

**
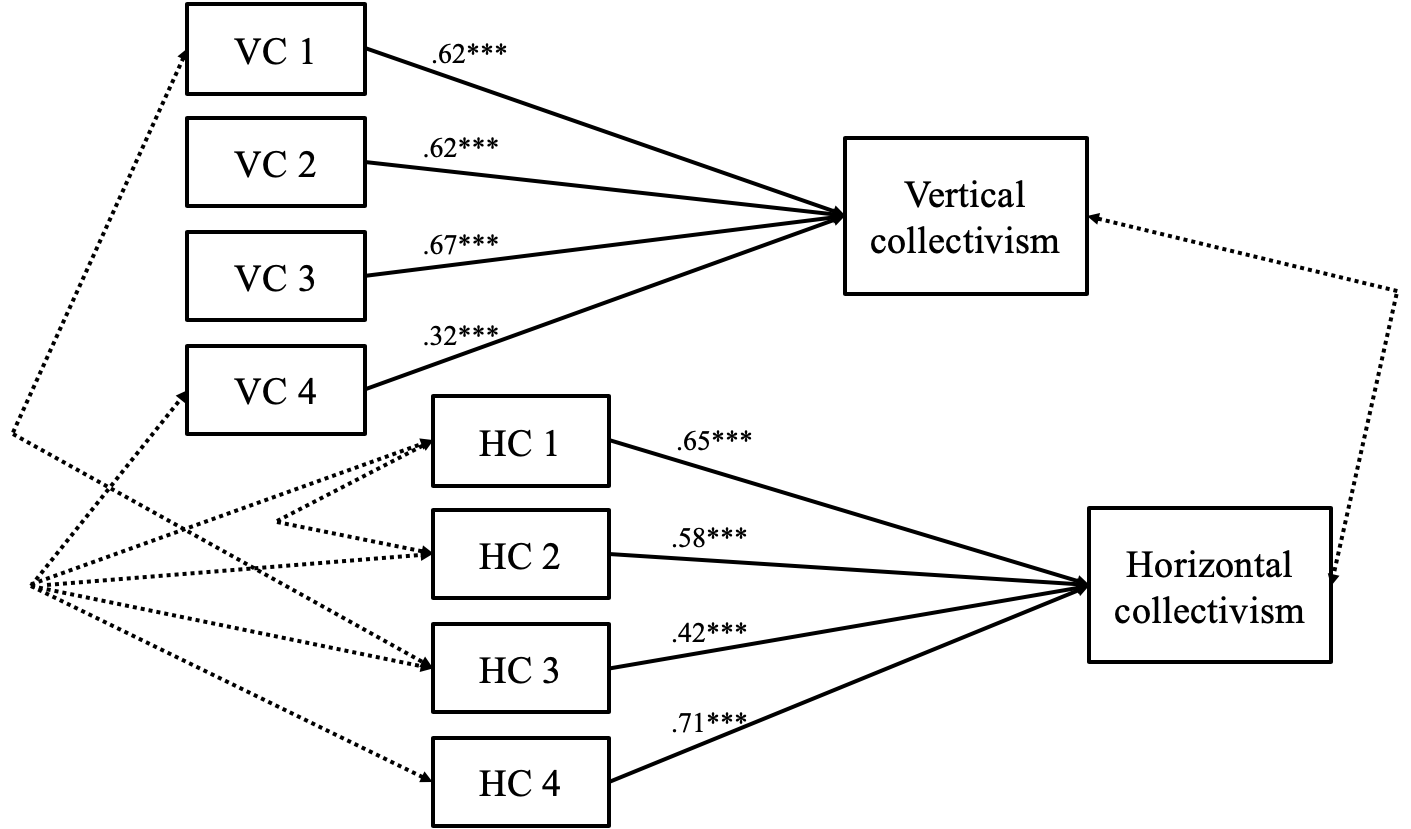
**

Global fit for collectivism CFA, χ^2^(13) = 22.77, *p* = .045; *CFI* = .99; *TLI* = .98; *RMSEA* = .03; *SRMR* = .02.

**Final model statistics**

|  | Hygiene | | Social distancing | | COVID-19 conspiracies | | Powerlessness | |
| --- | --- | --- | --- | --- | --- | --- | --- | --- |
| Variable | *B* (*SE*) | β | *B* (*SE*) | β | *B* (*SE*) | β | *B* (*SE*) | β |
| Vertical individualism | -0.02 (.02) | -.05 | -0.06 (.03) | -.12 | 0.13 (.03) | .24^***^ | 0.12 (.06) | .12^*^ |
| Vertical collectivism | - | - | 0.06 (.02) | .16^**^ | - | - | - | - |
| Horizontal collectivism | 0.04 (.02) | .12^*^ | -0.01 (.02) | -.02 | - | - | -0.19 (.05) | -.20^***^ |
| COVID-19 conspiracies | - | - | -0.15 (.04) | -.17^***^ | - | - | 0.37 (.09) | .18^***^ |
| Powerlessness | -0.09 (.02) | -.23^***^ | -0.09 (.02) | -.19^***^ | - | - |  |  |
| Political ideology | - | - | -0.06 (.03) | -.09^†^ | 0.11 (.03) | .15^**^ | - | - |
| Gender | 0.15 (.04) | .18^***^ | 0.24 (.05) | .23^***^ | 0.28 (.05) | .24^***^ | -0.31 (.10) | -.13^**^ |
| Education | - | - | - | - | -0.08 (.02) | -.15^***^ | - | - |
| Age | - | - | - | - | - | - | -0.01 (.01) | -.11^**^ |
| Religiosity | - | - | - | - | - | - | -0.07 (.03) | -.10^*^ |
| Health | -0.10 (.05) | -.10^*^ | -0.12 (.05) | -.10^*^ | - | - | - | - |

^†^*p* = .05, ^*^*p* < .05, ^**^*p* < .01, ^***^*p* < .001.

Political ideology: 1 = *extremely left wing*, 7 = *extremely right wing*. Gender: 1 = *Male,* 2 = *Female.* Health: 1 = *Yes to underlying health condition,* 2 = *No to underlying health condition.* Two paths are not included in this table: Vertical individualism positively predicting political ideology, *B* = 0.31, *SE* = .04, β = .42, *p* < .001, and vertical collectivism positively predicting religiosity, *B* = 0.35, *SE* = .06, β = .30, *p* < .001.

**Final model without controlling for demographics**


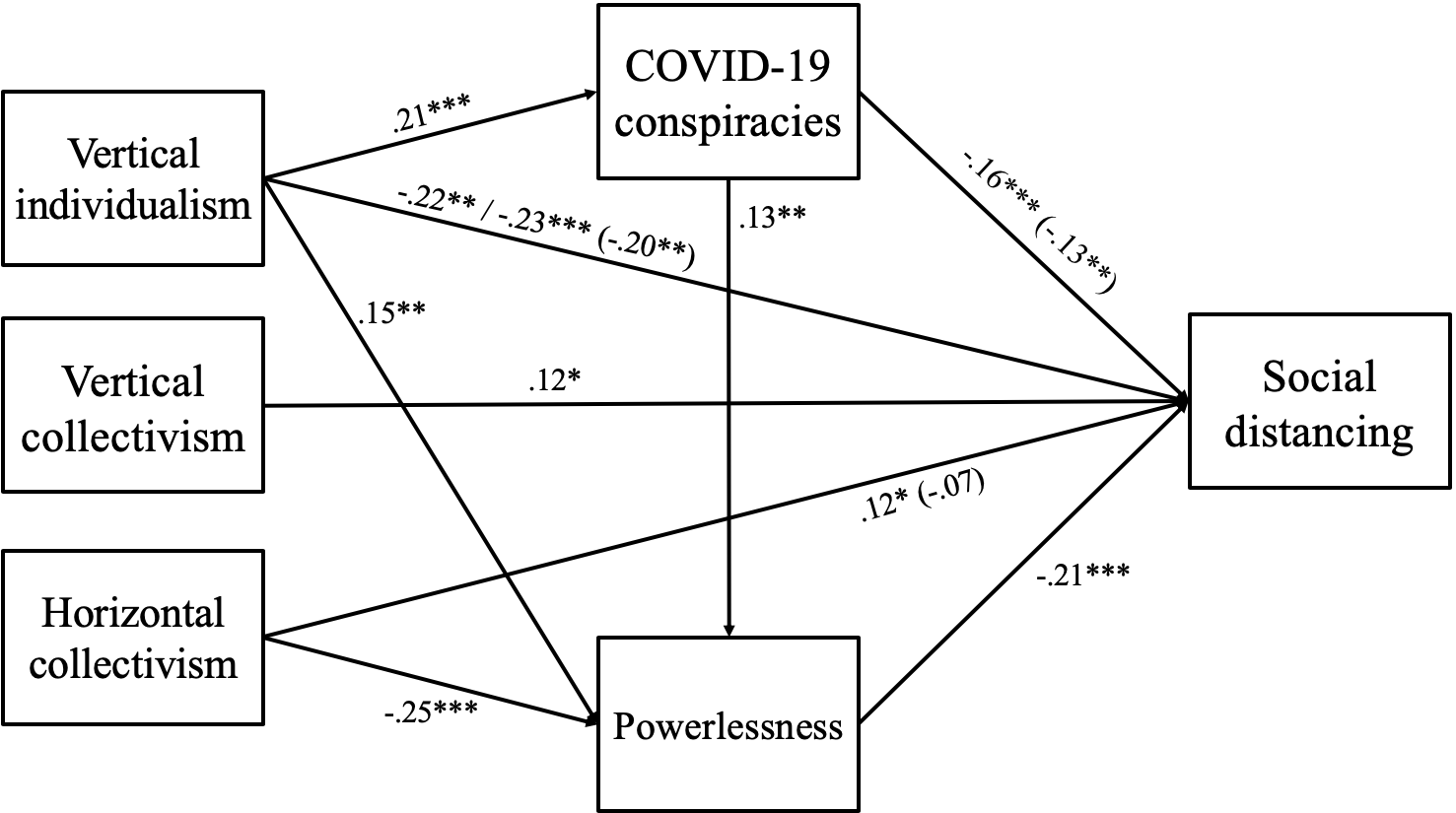


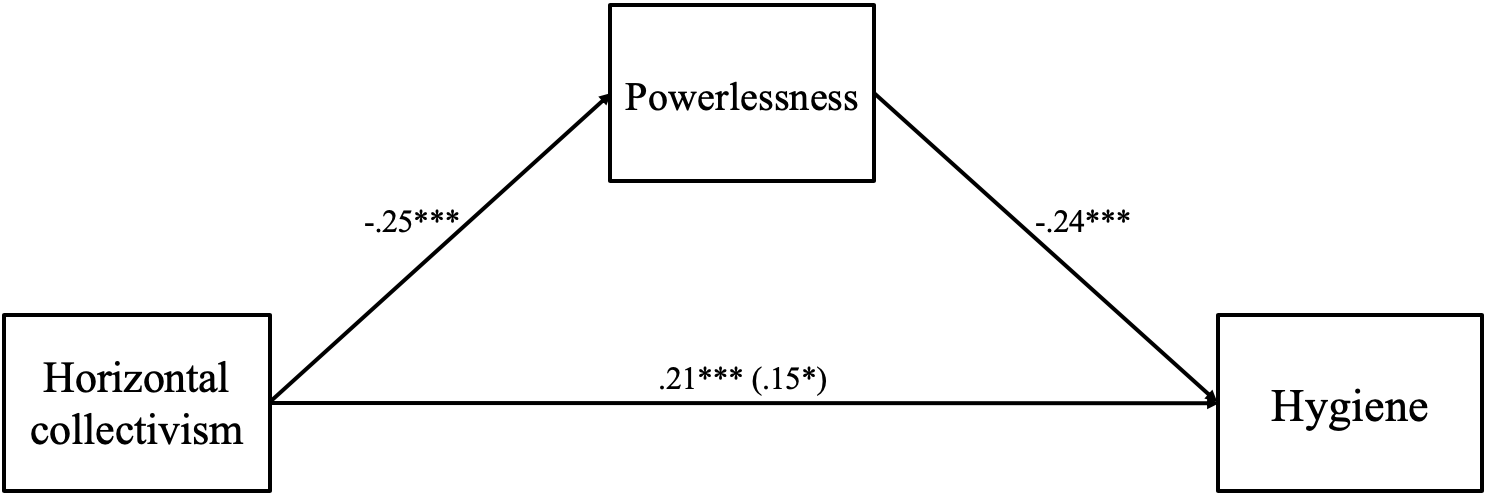


Model global fit, χ^2^(674) = 1385.98, *p* < .001; *CFI* = .93; *TLI* = .92; *RMSEA* = .04; *SRMR* = .05. Vertical individualism negatively predicted social distancing intentions through conspiracy belief, standardized indirect effect = -.03 [-0.04, -0.01] and powerlessness, standardized indirect effect = -.03 [-0.05, -0.01]. Horizontal collectivism positively predicted both social distancing intentions, standardized indirect effect = .05 [0.03, 0.07], and hygiene intentions, standardized indirect effect = .06 [0.04, 0.08], through powerlessness. The negative indirect effect of conspiracy belief on social distancing intentions through powerlessness had confidence intervals that included zero.

**Final model without the path from conspiracy belief to powerlessness**


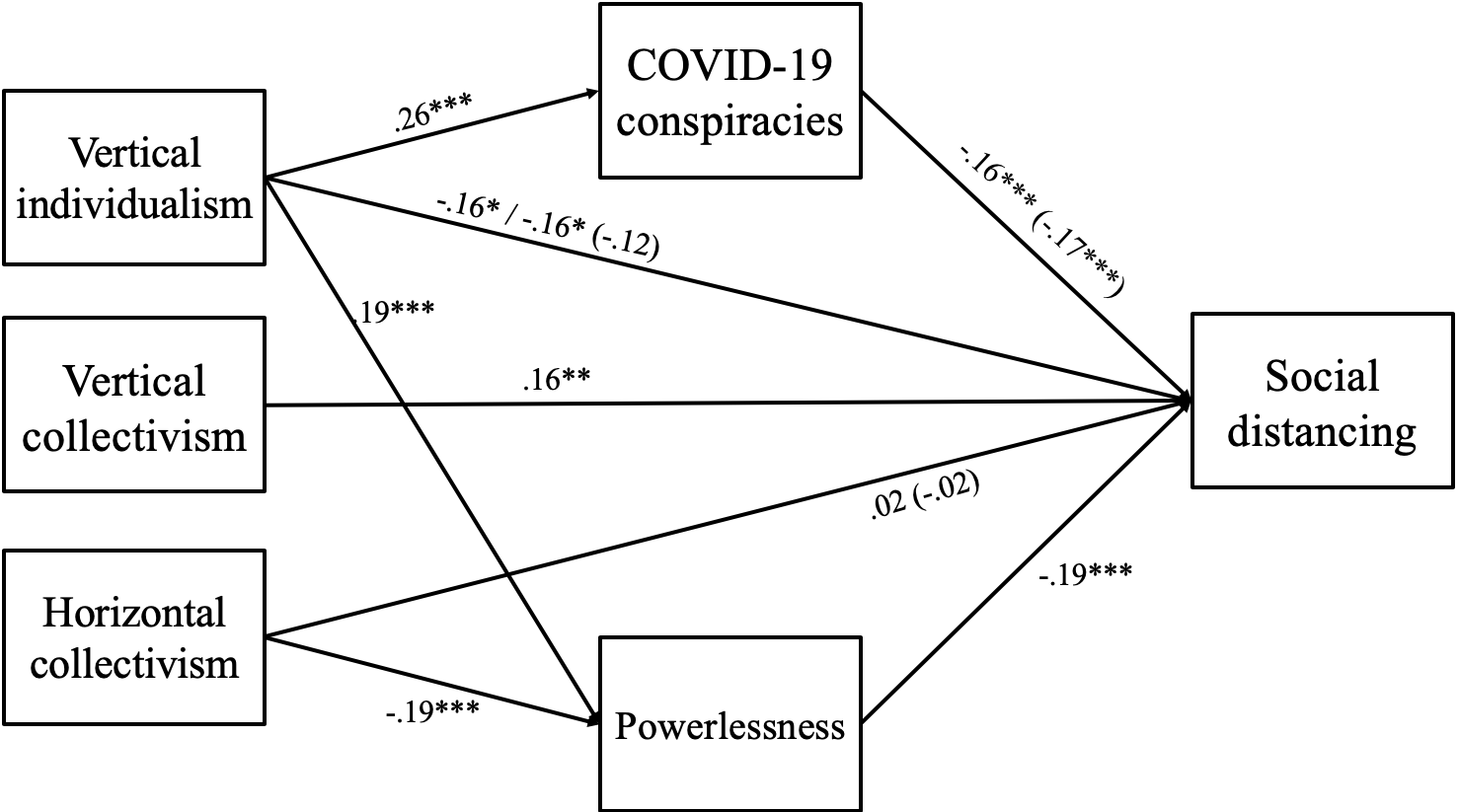


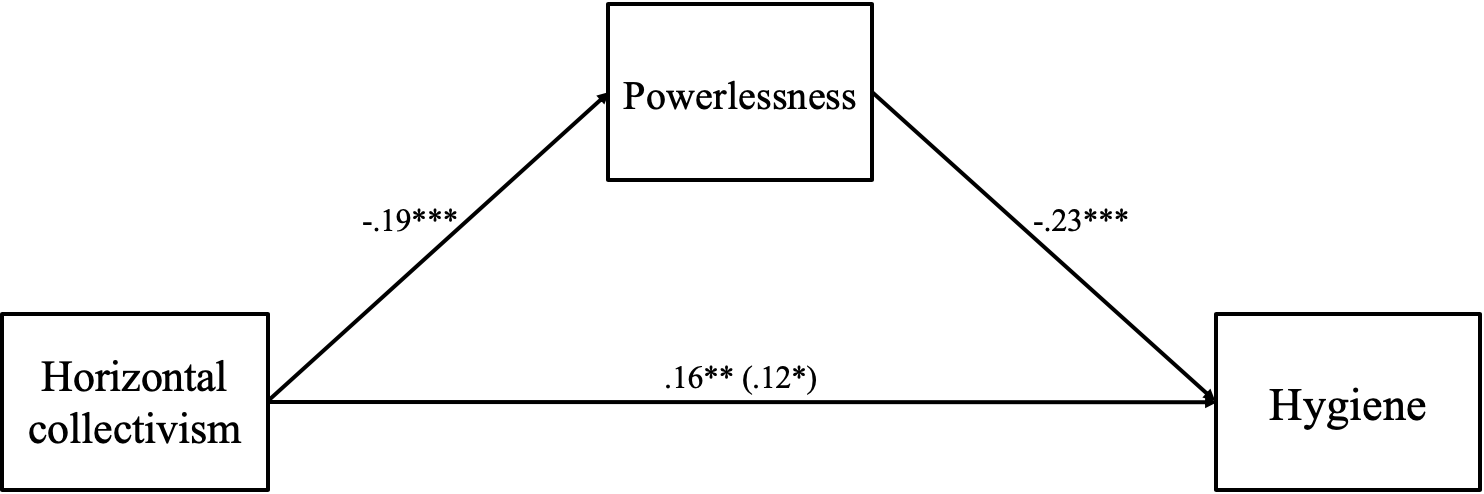


Model global fit, χ^2^(905) = 2031.52, *p* < .001; *CFI* = .89; *TLI* = .88; *RMSEA* = .04; *SRMR* = .06. Vertical individualism negatively predicted social distancing intentions through both conspiracy belief, standardized indirect effect = -.05 [-0.07, -0.02], and powerlessness, standardized indirect effect = -.04 [-0.06, -0.02]. Horizontal collectivism positively predicted both social distancing, standardized indirect effect = .04 [0.02, 0.06], and hygiene intentions, standardized indirect effect = .04 [0.02, 0.07] through powerlessness.

**Final model controlling for nationality (UK and USA participants vs. Other)**


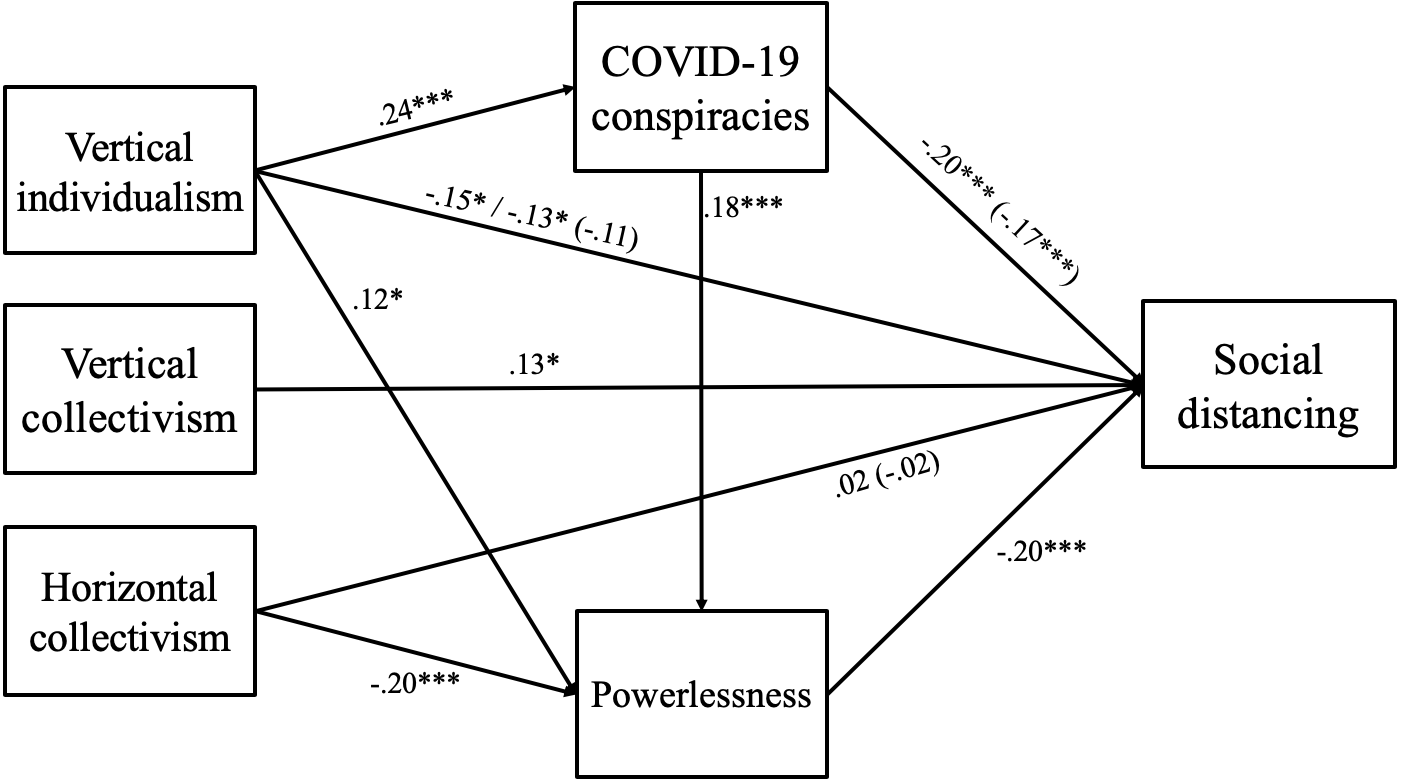


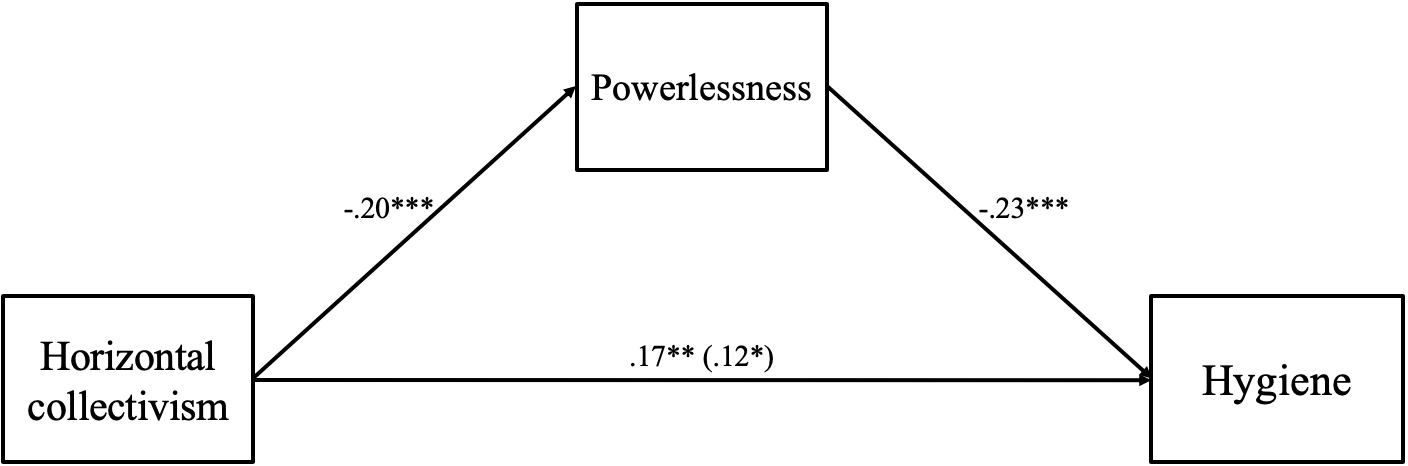


Model global fit, χ^2^(939) = 2095.63, *p* < .001; *CFI* = .88; *TLI* = .87; *RMSEA* = .04; *SRMR* = .06. UK and USA participants comprised the majority of the sample, so we tested the model controlling for nationality (UK/USA or Other). Vertical individualism negatively predicted social distancing through conspiracy belief, standardized indirect effect = -.04 [-0.06, -0.02], and powerlessness, standardized indirect effect = -.02 [-0.05, -0.01]. Horizontal collectivism positively predicted social distancing through powerlessness, standardized indirect effect = .04 [0.02, 0.06]. Horizontal collectivism positively predicted hygiene intentions through powerlessness, standardized indirect effect = .05 [0.02, 0.07]. Finally, conspiracy belief did not negatively predicted social distancing intentions through powerlessness, standardized indirect effect = -.04 [-0.07, 0.01].

**Final model controlling for sample source**

**
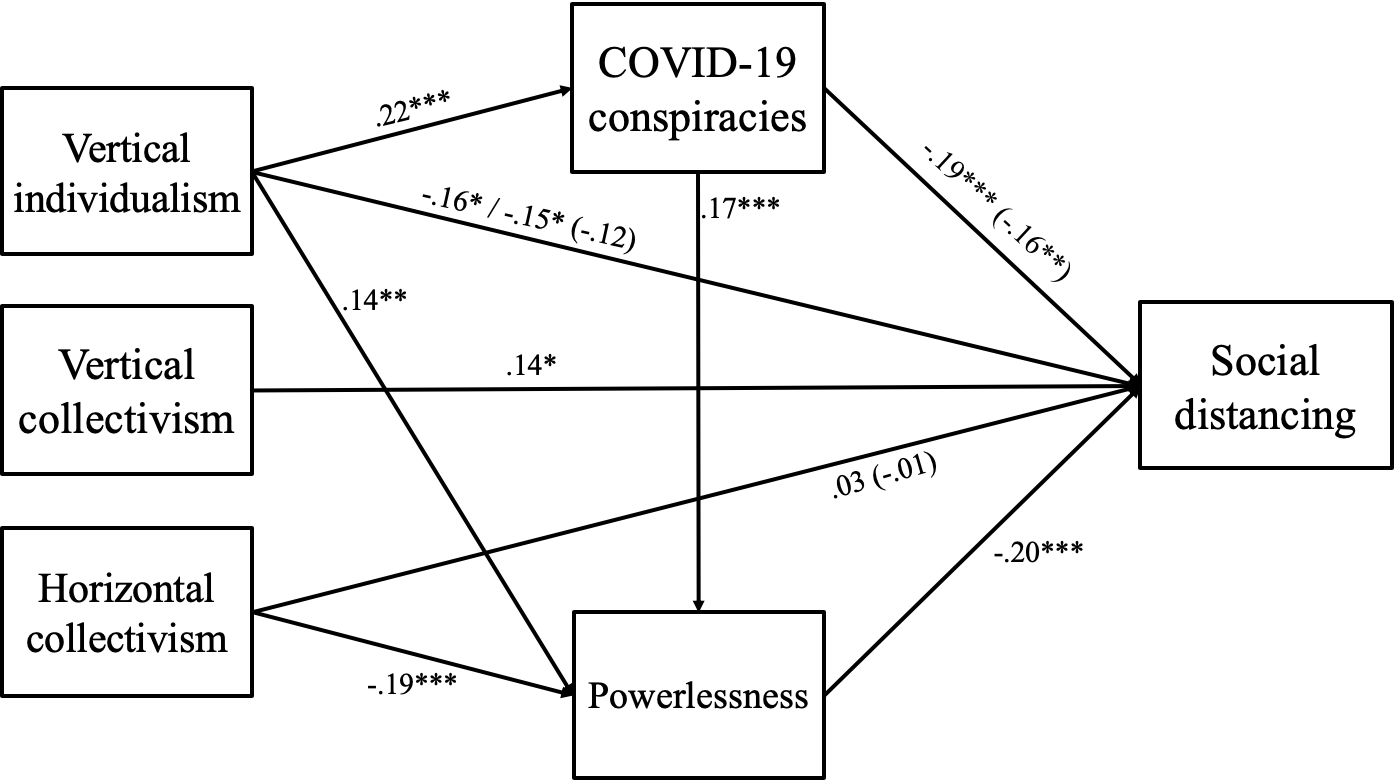
**

**
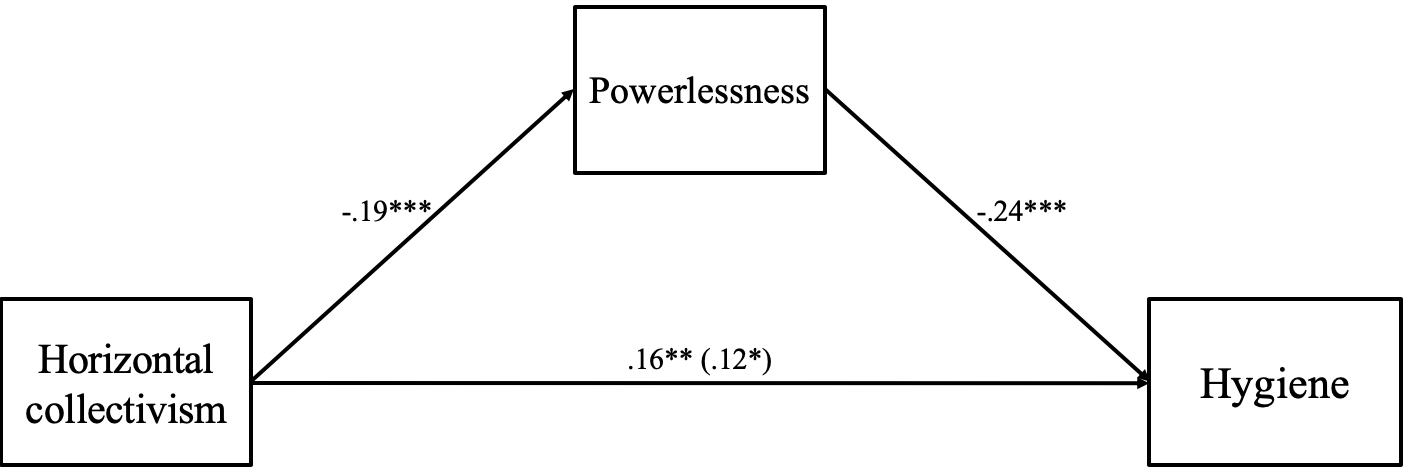
**

Model global fit, χ^2^(980) = 2174.72, *p* < .001; *CFI* = .88; *TLI* = .87; *RMSEA* = .04; *SRMR* = .06. We also controlled for the source of data collection (Social media versus Reddit). Vertical individualism negatively predicted social distancing intentions through conspiracy belief, standardized indirect effect = -.04 [-0.06, -0.02], and powerlessness, standardized indirect effect = -.03 [-0.05, -0.01]. Horizontal collectivism positively predicted social distancing intentions through powerlessness, standardized indirect effect = .04 [0.02, 0.06], and hygiene intentions through the same mediator, standardized indirect effect = .05 [0.02, 0.07]. Conspiracy belief did not negatively predict social distancing intentions through powerlessness, standardized indirect effect = -.03 [-0.07, 0.01].

**Study materials**

***Intentions***

“Please indicate how likely you are to engage in the following behaviours in the next month, from 1 (definitely not) to 5 (definitely yes).”

1. Wash your hands after using the toilet
2. Wash your hands after every outing
3. Wash your hands before eating
4. Remain at least 2 meters (6 feet) away from other people
5. Scratch an itch on your face (reverse coded)
6. Shake someone's hand to greet them (reverse coded)
7. Hug a friend or loved one to greet them (reverse coded)
8. Kiss a friend or loved one (reverse coded)
9. Isolate yourself for at least 1 week if you show even mild cold or flu symptoms
10. Isolate yourself for at least 1 week if you know you have been in contact with someone with Coronavirus
11. Attend a social gathering
12. Board a flight in order to attend the funeral of a loved one

Attention check: Please indicate the fourth option "Probably yes" to show you are paying attention

***COVID-19 conspiracy theories***

**“**Please indicate your agreement with the following statements, from 1 (strongly disagree) to 5 (strongly agree).”

1. A lot of information about Coronavirus is deliberately held back from the public
2. There is a cure for Coronavirus, but it is being withheld by the government
3. Coronavirus was created and spread by the CIA
4. Coronavirus was created by the government to control the population
5. The existence of Coronavirus is a hoax perpetuated by the media
6. The implementation of 5G technology is a means of deliberately spreading Coronavirus
7. Coronavirus was purposefully created in, and released from, a biochemistry lab in Wuhan, China
8. The US government purposefully released Coronavirus into the Chinese population to severely hurt its economic growth
9. Pharmaceutical companies created and released Coronavirus in order to sell their medications and vaccines
10. The new world order have finally found their most effective means of controlling populations through the release of Coronavirus

Attention check: Please choose the second option "disagree" to show you are paying attention

***Powerlessness*** (adapted from Jolley & Douglas, 2014a, 2014b)

“Please indicate your agreement with the following statements about the spread of Coronavirus, from 1 (strongly disagree) to 6 (strongly agree).”

1. I feel that the Coronavirus is too big for my actions to have an impact
2. I feel that my actions will not affect the outcome of Coronavirus
3. I feel that my contribution is just a drop in the ocean and so is insignificant

***Cultural orientation*** (Triandis & Gelfand, 1998)

“Please indicate how well each of the following statements describe yourself, from 1 (definitely no) to 9 (definitely yes).”

Horizontal individualism items:

1. I'd rather depend on myself than others.

2. I rely on myself most of the time; I rarely rely on others.

3. I often do "my own thing."

4. My personal identity, independent of others, is very important to me.

Vertical individualism items:

1. It is important that I do my job better than others.

2. Winning is everything.

3. Competition is the law of nature.

4. When another person does better than I do, I get tense and aroused.

Horizontal collectivism items:

1. If a coworker gets a prize, I would feel proud.

2. The well-being of my coworkers is important to me.

3. To me, pleasure is spending time with others.

4. I feel good when I cooperate with others.

Vertical collectivism items:

1. Parents and children must stay together as much as possible.

2. It is my duty to take care of my family, even when I have to sacrifice what I want.

3. Family members should stick together, no matter what sacrifices are required.

4. It is important to me that I respect the decisions made by my groups.

**All variables included in the pre-registration**

We ran a model with all of the paths mentioned in our pre-registration. Below are all of the significant effects that were obtained in this analysis. This model had poor global fit, χ^2^ = 851.95, *p* < .001; *CFI* = .48; *TLI* = .25; *RMSEA* = .12, *p* < .001; *SRMR* = .09, but was not intended for use in the final analysis. Missing from the tables are the effects of horizontal collectivism positively predicting blaming the self, *B* = 0.09, *SE* = .04, β = .08, [.01, .16], *p* = .026, and risk, *B* = 0.11, *SE* = .02, β = .24, [.08, .15], *p* < .001, alongside belief in COVID-19 conspiracy theories positively predicted uncertainty, *B* = 0.81, *SE* = .07, β = .40, [.67, .95], *p* < .001, and vertical individualism positively predicting belief in COVID-19 conspiracy theories, *B* = 0.05, *SE* = .01, β = .14, [.03, .08], *p* < .001.

|  |  |  | Hygiene |  |
| --- | --- | --- | --- | --- |
| Variable | *B* (*SE*) | β | 95% CI | *p* |
| Powerlessness | -0.09 (.02) | -.18 | [-.13, -.05] | < .001 |
| Blaming governments | -0.06 (.02) | -.10 | [-.10, -.02] | .008 |
| Blaming behaviours | 0.08 (.02) | .13 | [.03, .12] | .001 |
| Blaming citizens | 0.05 (.02) | .09 | [.01, .09] | .008 |
| Risk | 0.22 (.04) | .23 | [.15, .29] | < .001 |
| Communal orientation | -0.11 (.04) | -.09 | [-.18, -.03] | .009 |
| Uncertainty | -0.04 (.02) | -.07 | [-.08, -.01] | .042 |

|  | Social distancing | | | |
| --- | --- | --- | --- | --- |
| Variable | *B* (*SE*) | β | 95% CI | *p* |
| COVID-19 conspiracies | -0.15 (.04) | -.13 | [-.22, -.07] | < .001 |
| Powerlessness | -0.09 (.02) | -.17 | [-.13, -.06] | < .001 |
| Horizontal collectivism | 0.04 (.02) | .07 | [.01, .07] | .045 |
| Blaming behaviours | 0.07 (.02) | .13 | [.03, .11] | < .001 |
| Risk | 0.20 (.04) | .19 | [.12, .27] | < .001 |
| Uniqueness | -0.02 (.01) | -.09 | [-.04, -.01] | .007 |

|  | Powerlessness | | | |
| --- | --- | --- | --- | --- |
| Variable | *B* (*SE*) | β | 95% CI | *p* |
| Vertical individualism | 0.10 (.03) | .12 | [.04, .15] | .001 |
| Horizontal collectivism | -0.22 (.03) | -.24 | [-.28, -.15] | < .001 |
| COVID-19 conspiracies | 0.33 (.08) | .16 | [.18, .48] | < .001 |

|  | Trust | | | |
| --- | --- | --- | --- | --- |
| Variable | *B* (*SE*) | β | 95% CI | *p* |
| Horizontal collectivism | 0.09 (.02) | .16 | [.05, .13] | < .001 |
| COVID-19 conspiracies | -0.44 (.04) | -.35 | [-.53, -.35] | < .001 |

|  | Uniqueness | | | |
| --- | --- | --- | --- | --- |
| Variable | *B* (*SE*) | β | 95% CI | *p* |
| Vertical individualism | 0.35 (.07) | .19 | [.21, .49] | < .001 |
| Vertical collectivism | -0.24 (.07) | -.12 | [-.38, -.09] | .001 |

|  | Communal orientation | | | |
| --- | --- | --- | --- | --- |
| Variable | *B* (*SE*) | β | 95% CI | *p* |
| Vertical individualism | 0.08 (.01) | .24 | [.06, .11] | < .001 |
| Horizontal collectivism | -0.04 (.02) | -.11 | [-.07, -.02] | .005 |

|  | Blaming citizens | | | |
| --- | --- | --- | --- | --- |
| Variable | *B* (*SE*) | β | 95% CI | *p* |
| Vertical individualism | 0.07 (.03) | .10 | [.02, .13] | .006 |
| COVID-19 conspiracies | 0.30 (.07) | .16 | [.16, .45] | < .001 |
